# Dynamics of time-resolved photoluminescence in GaInNAs and GaNAsSb solar cells

**DOI:** 10.1186/1556-276X-9-80

**Published:** 2014-02-17

**Authors:** Alexander Gubanov, Ville Polojärvi, Arto Aho, Antti Tukiainen, Nikolai V Tkachenko, Mircea Guina

**Affiliations:** 1 Optoelectronics Research Centre, Tampere University of Technology, P.O. Box 692, Tampere FIN-33101, Finland; 2 Department of Chemistry and Bioengineering, Tampere University of Technology, P.O. Box 541, Tampere FIN-33101, Finland

**Keywords:** Solar cells, Dilute nitrides, GaInNAsSb, Time-resolved photoluminescence, Carrier lifetime

## Abstract

We report a time-resolved photoluminescence study for GaInNAs and GaNAsSb p-i-n bulk solar cells grown on GaAs(100). In particular, we studied the extent to which the carrier lifetime decreases with the increase of N content. Rapid thermal annealing proved to significantly increase the decay times by a factor of 10 to 12 times, for both GaInNAs and GaNAsSb heterostructures, while for the 1-eV bandgap GaNAsSb structure, grown at the same growth conditions as the GaInNAs, the photoluminescence decay time remained slightly below 100 ps after annealing; the approximately 1.15-eV GaInNAs p-i-n solar cell exhibited a lifetime as long as 900 ps.

**PACS:**

78.47.D; 78.55.Cr; 88.40.hj

## Background

In recent years, multijunction III-V semiconductor solar cells have experienced remarkable improvements, not only for space applications but also for terrestrial concentrated photovoltaic systems. The highest photovoltaic conversion efficiency reported so far is 44.7% and has been obtained with four junction solar cell [1]. A very promising way to further improve the performance of solar cells is to utilize dilute nitride and dilute antimonide materials, which can be grown lattice matched onto GaAs and Ge substrates [2]. These materials provide suitable absorption bands to harvest photons down to 1 eV and even below. Recently, a conversion efficiency of 44% was reported for a triple junction solar cell including a bottom junction based on GaInNAs(Sb) grown by molecular beam epitaxy (MBE) [3]. Adding antimony to ternary GaAsN to form GaAsNSb compounds can be also used to lower the bandgap beyond the 1-eV limit, serving as an alternative to quinary alloys, which are somewhat more difficult to grow due to the presence of three elements of group V [4,5]. The drawback in using dilute nitrides/antimonides is related to challenges in material fabrication [6] and formation of defects [7,8]. Careful growth parameter optimization and thermal annealing are known to increase the material quality and carrier lifetimes [9]. Carrier lifetime correlates with solar cell performance via the minimum diffusion length required for the carriers to travel without recombination, and it should be maximized in order to harvest efficiently the photogenerated carriers [10]. Time-resolved photoluminescence (TRPL) using up-conversion technique [11] is commonly used for estimating carrier lifetimes of optoelectronic heterostructures and has been extensively used in connection with optimization of GaInNAs heterostructures [2,12-14]. However, most of the studies have been concerned with analyses of quantum wells [15]. Studies on GaInAsN epilayers have reported a wide variety of lifetimes in the range of 70 to 740 ps [8,16]. In this paper, we report TRPL values for bulk GaInAsN and GaNAsSb p-i-n solar cells. In particular, we focus on correlating the effects of thermal annealing and the nitrogen composition.

## Methods

**Figure 1:**
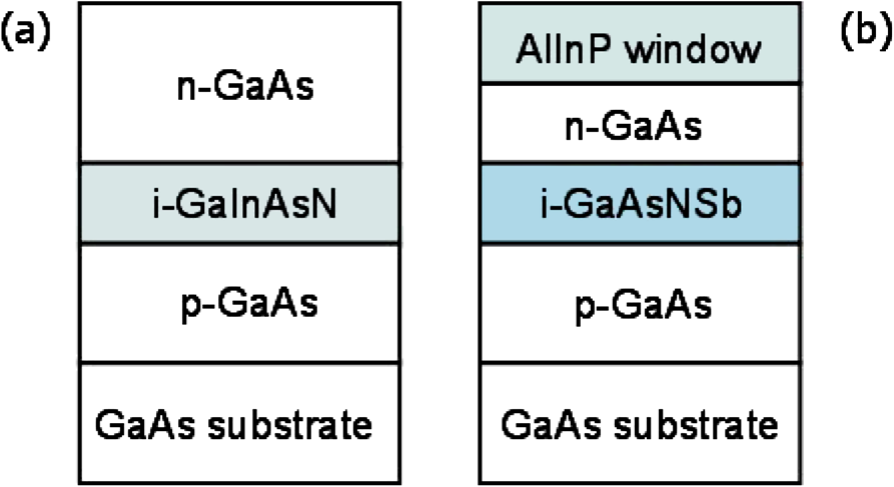
**Schematic sample structures for (a) samples 1, 2, 3, and (b) sample 4.** The thickness of the lattice-matched N-based intrinsic regions is ranging from 300 to 1,300 nm.

The samples studied were grown on GaAs(100) substrate by MBE equipped with radio-frequency plasma source for atomic nitrogen incorporation. Their structures are presented in Figure 1. The thickness of the intrinsic region of the p-i-n solar cells grown was modified throughout the series, but other growth parameters were kept constant. The intrinsic regions of samples 1, 2, and 3 consist of lattice-matched GaInNAs with nitrogen compositions of 1%, 2%, and 3%, and were 320-, 600-, and 600-nm thick, respectively. In order to obtain lattice matching, the In composition was 2.7 times the nitrogen composition in each of the samples. Sample 4 comprised a lattice-matched GaN_0.02_As_0.93_Sb_0.05_ intrinsic region with a bandgap of approximately 1 eV and, unlike the other samples, had also an AlInP window layer. After growth, wafers were diced and thermally annealed. Rapid thermal annealing (RTA) treatments were done in N_2_ atmosphere. Sample temperature was monitored by optical pyrometer through the Si carrier wafer. In order to avoid desorption of As, the samples were protected with a GaAs proximity cap during RTA [17]. The annealing temperatures and the corresponding times for samples 1 to 3 were optimized to maximize the PL intensity [18].

TRPL measurements were carried out with an up-conversion system [19]. For instrumentation details, see [20]. The excitation source was an 800-nm mode-locked Ti-sapphire pulsed laser, which delivered 50-fs pulses enabling a final time resolution of approximately 200 fs (FWHM). The excitation density was approximately 3 × 10^-4^ J/cm^2^, with a 20-μm diameter spot on the sample.

The population dynamics of a single radiative level is given by a rate equation:

(1) $$\frac{\mathrm{dn}\left(t\right)}{\mathrm{dt}}=-k\times n\left(t\right),$$

which results in a monoexponential photoluminescence decay [21]:

(2) $$n\left(t\right)=A\exp\;\left(-t/\tau_{\text{decay}}\right).$$

This model ignores thermalization of carriers after excitation, which is typically a very fast process and was not time-resolved in these measurements. To account for limited time resolution of the instrument, emission decays were fitted using deconvolution with the instrument response function. The monoexponential fits gave satisfactory results for all measured decays.

## Results and discussion

**Figure 2:**
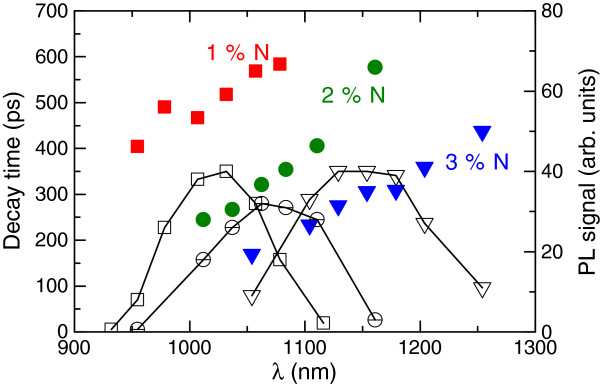
Wavelength dependences of decay time constants for samples 1-3 with GaInAsN i-region and PL intensities.

Figure 2 shows the fit results for TRPL data for samples 1 to 3 measured at different wavelengths. Emission wavelength depends on the nitrogen and indium composition, as shown by lines and open points in Figure 2. The photoluminescence emission spectra appear to be rather broad, which is typical for bulk-like heterostructures. The decay time increased steadily with the wavelength, being within 400 to 600 ps for sample 1 and in 200 to 400 ps range for samples 2 and 3.

The spectral dependence of carrier lifetime in GaInNAs can be explained in terms of interplay between the radiative recombination and hopping energy relaxation of localized excitons as described by Rubel et al. [22] and references therein. According to Takahashi et. al [23], the increased nitrogen concentration, as in samples 1 to 3, merely increases the band bowing and reduces the dipole interaction by involving more non-Γ states, which has a direct effect on the radiative lifetime when larger N concentrations are used. Moreover, nitrogen increases the density of nonradiative recombination centers in the bandgap which strongly contributes to the carrier lifetime.

**Figure 3:**
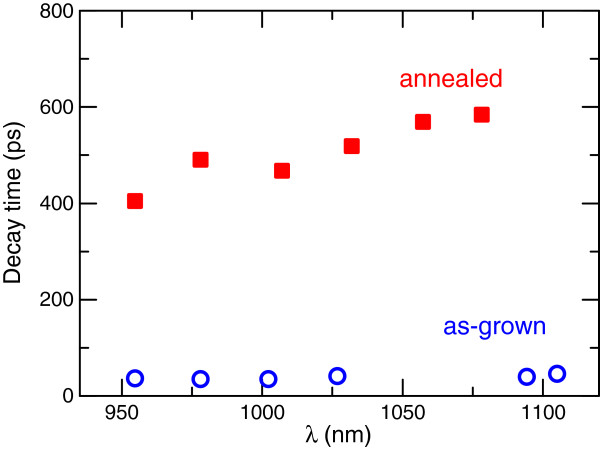
Decay time versus wavelength for as-grown and annealed sample 1.

Annealing indeed increases the decay time of GaInNAs, and this is shown in Figure 3, where the as-grown sample decay time is also plotted. Lifetime increases by one order of magnitude following RTA, underlining the importance of thermal annealing for dilute nitride solar cells. Optimal annealing conditions for GaInNAs depend on the amount of nitrogen and growth parameters. Typically, good results for solar cells are obtained when annealing is performed at 750°C to 800°C for a few hundred seconds [24,25]. This significant increase of decay time is related to reduction of nonradiative recombination and removal of defects due to thermal annealing [26,27]. Furthermore, the decrease of decay times for the higher nitrogen content points out to the fact that that nitrogen-related defects are responsible for decreasing the carrier lifetime [13].

**Figure 4:**
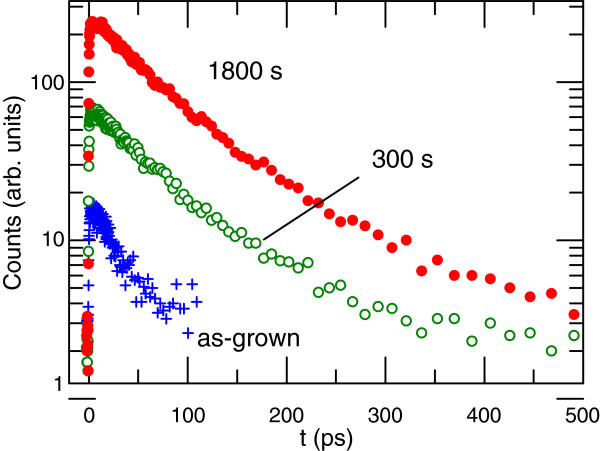
**Decay profiles for sample 4 comprising GaNAsSb measured at *λ*** **= 1,250 nm.** Annealing time at *T*_ann_ = 750°C was 0, 300, and 1,800 s.

**Figure 5:**
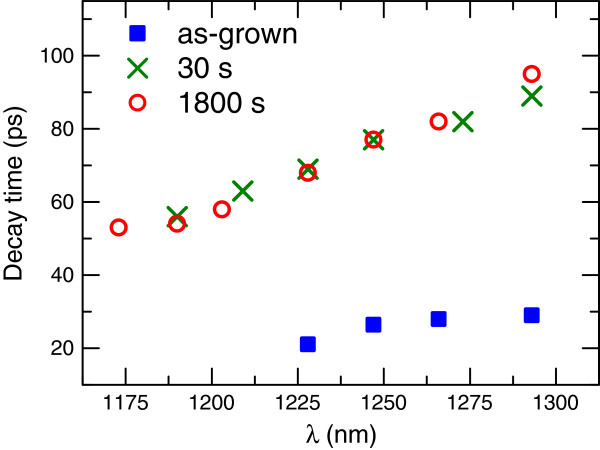
**Wavelength-dependent decay times *τ *for sample 4 with GaNAsSb i-region.** Annealed at *T*_ann_ = 750°C for 0, 30, and 1,800 s.

The effect of RTA was further investigated on the GaNAsSb structure. Figure 4 shows TRPL decays for sample 4 for as-grown wafer and annealing times of 300 and 1,800 s at a temperature (*T*_ann_) of 750°C. The dependences of decay time on detection wavelength are presented in Figure 5. An increase in decay time is observed when moving towards the band edge, which is similar to samples 1 to 3. The change in the *τ*(*λ*) slope upon RTA can be linked to carrier energy relaxation processes in the vicinity of the conduction band edge [22]. Although lifetime increases with annealing, it remained below 100 ps. Furthermore, sample 4 has AlInP window layer which suppresses effectively surface recombination rates. This lifetime is approximately one fourth of that for sample 3 and one half of the value obtained for the quinary GaInNAsSb [8]. Furthermore, as high as 900 ps, lifetime (not shown) was measured from an optimized GaInNAs p-i-n solar cell structure with an approximately 1.15-eV bandgap [9]. The fact that the lifetime after annealing is one order of magnitude less than for optimized GaInNAs and less than what has been published for GaInNAsSb indicates that there is still room for further optimization for GaNAsSb growth and annealing parameters.

## Conclusions

We investigated the carrier lifetime dynamics in lattice-matched GaInNAs and GaNAsSb p-i-n solar cells using TRPL. The increase of nitrogen content decreases the carrier lifetime owing to increase of defect densities. An increase of the lifetime by at least tenfold was observed after thermal annealing of bulk GaInNAs layers. Thermal annealing was also found to affect the carrier energy relaxation process in GaNAsSb. Further growth and annealing parameter optimization is needed to improve the quality of GaNAsSb to make it an effective subjunction material in high-efficiency terrestrial and space solar cells.

## Competing interests

The authors declare that they have no competing interests.

## Authors’ contributions

The samples were fabricated under the supervision of AA and AT. Post growth sample preparation was supervised by VP. The experimental part was performed by AG and NVT, the numerical calculation was carried out by AG, and the manuscript was written by VP, AG, AT, and MG. All authors read and approved the final manuscript.
